# Synaptic Vesicles Having Large Contact Areas with the Presynaptic Membrane are Preferentially Hemifused at Active Zones of Frog Neuromuscular Junctions Fixed during Synaptic Activity

**DOI:** 10.3390/ijms20112692

**Published:** 2019-05-31

**Authors:** Jae Hoon Jung

**Affiliations:** 1 Department of Biology, Texas A&M University, College Station, TX 77845, USA; jjung@bio.tamu.edu; 2 Department of Physics, Stanford University School of Humanities and Sciences, Stanford, CA 94309, USA

**Keywords:** electron tomography, active zone, synaptic vesicle, hemifusion, synaptic transmission, nerve terminal, synapse

## Abstract

Synaptic vesicles dock on the presynaptic plasma membrane of axon terminals and become ready to fuse with the presynaptic membrane or primed. Fusion of the vesicle membrane and presynaptic membrane results in the formation of a pore between the membranes, through which the vesicle’s neurotransmitter is released into the synaptic cleft. A recent electron tomography study on frog neuromuscular junctions fixed at rest showed that there is no discernible gap between or merging of the membrane of docked synaptic vesicles with the presynaptic membrane, however, the extent of the contact area between the membrane of docked synaptic vesicles and the presynaptic membrane varies 10-fold with a normal distribution. The study also showed that when the neuromuscular junctions are fixed during repetitive electrical nerve stimulation, the portion of large contact areas in the distribution is reduced compared to the portion of small contact areas, suggesting that docked synaptic vesicles with the largest contact areas are greatly primed to fuse with the membrane. Furthermore, the finding of several hemifused synaptic vesicles among the docked vesicles was briefly reported. Here, the spatial relationship of 81 synaptic vesicles with the presynaptic membrane at active zones of the neuromuscular junctions fixed during stimulation is described in detail. For the most of the vesicles, the combined thickness of each of their contact sites was not different from the sum of the membrane thicknesses of the vesicle membrane and presynaptic membrane, similar to the docked vesicles at active zones of the resting neuromuscular junctions. However, the combined membrane thickness of a small portion of the vesicles was considerably less than the sum of the membrane thicknesses, indicating that the membranes at their contact sites were fixed in a state of hemifusion. Moreover, the hemifused vesicles were found to have large contact areas with the presynaptic membrane. These findings support the recently proposed hypothesis that, at frog neuromuscular junctions, docked synaptic vesicles with the largest contact areas are most primed for fusion with the presynaptic membrane, and that hemifusion is a fusion intermediate step of the vesicle membrane with the presynaptic membrane for synaptic transmission.

## 1. Introduction

Synaptic vesicles are docked or held at specialized regions, called active zones, on the presynaptic plasma membrane [1,2]. In the vicinity of the synaptic vesicles at active zones there are aggregates of macromolecules bound to the presynaptic membrane, called active zone material (AZM), [1,3] and clusters of voltage-gated Ca^2+^ channels [4,5,6]. After a nerve impulse arrival, the Ca^2+^ channels open and synaptic vesicles, which are fusion-ready or primed, near the channels fuse with the presynaptic membrane and release the neurotransmitter to elicit the postsynaptic response for synaptic transmission [7,8]. The Ca^2+^-triggered synaptic vesicle fusion with the presynaptic membrane is mediated by N-ethylmaleimide-sensitive factor activation protein receptor (SNARE) proteins and their auxiliary proteins [9,10,11,12,13,14,15,16,17,18,19,20,21]. The fusion pore between the vesicle membrane and the presynaptic membrane is generally thought to be formed by the distortion of the vesicle membrane and the presynaptic membrane in contact, which depends on the force generated by the formation of SNARE complex [22,23]. Consistently, a significant number of studies using reconstituted liposomes have shown that the fusion of the liposomes can be mediated by SNARE proteins and their auxiliary proteins. Furthermore, the hemifusion state in which two membranes in contact merge into one membrane has also been observed [24,25,26,27,28,29,30,31,32], which is thought to promote membrane fusion by lowering the energy barrier for the formation of lipidic fusion pore. Despite the hemifusion state being widely accepted as a fusion intermediate [33], hemifused synaptic vesicles with the presynaptic membrane at the active zone have not been reported, except for only a few studies [34,35]. Furthermore, several studies suggested proteinaceous fusion pore formation, which does not require hemifusion [36,37,38,39,40]. The studies on hemifused synaptic vesicles at active zones have relied on electron tomography because it can provide sufficient spatial resolution to discern hemifused membranes from membranes in contact without such membrane merging. Electron tomography studies on brain slices of rats reported that at nerve terminals, a significant portion of the docked synaptic vesicles at active zones are hemifused [34,35]. In contrast, other electron tomography studies on hippocampal slices of rats and mice reported no evidence of hemifused synaptic vesicles [41,42,43,44]. Consistently, previous studies on resting frog neuromuscular junctions (NMJs) using electron tomography found no evidence of hemifused synaptic vesicles at active zones, raising some concerns as to whether synaptic vesicles go through hemifusion prior to fusion with the presynaptic membrane. Alternatively, synaptic vesicles might have hemifusion-independent membrane fusion pathways.

A recent electron tomography study explored synaptic vesicles at active zones of axon terminals in frog NMJs, chemically fixed at rest and during electrical stimulation at 10 Hz [45]. This chemical fixation method has been used to capture synaptic vesicles undergoing a variety of processes during evoked synaptic activity; synaptic vesicles at active zones are found to be docked, undocked, or fused with the presynaptic membrane [3,46,47]. Using electron tomography, which provides 2–3 nm spatial resolution [48], Jung et al. (2016) examined the spatial relationship of synaptic vesicles with the presynaptic membrane at active zones fixed at rest and during the electrical stimulation [45], and it was discovered that a small portion of docked synaptic vesicles at active zones fixed during the electrical stimulation were hemifused with the presynaptic membrane, whereas there were no hemifused synaptic vesicles at active zones fixed at rest. Moreover, their contact sites were quantitatively characterized by measuring the membrane thicknesses of vesicles and the presynaptic membranes away from their contact sites, the combined membrane thicknesses at their contact sites, and their contact areas. The measured combined membrane thickness at the contact site between each of the hemifused synaptic vesicles and the presynaptic membrane showed a significantly smaller thickness than the sum of the two membrane thicknesses. Furthermore, it was also noted that the contact areas of the hemifused synaptic vesicles were similar to those large contact areas of docked synaptic vesicles at resting active zones [45]. Here, the spatial relationship of the docked and hemifused synaptic vesicles at the active zones of the junctions fixed during stimulation was quantitatively detailed further. All the docked synaptic vesicles without hemifusion showed that their combined membrane thickness was not different from the sum of the vesicle and presynaptic membrane thicknesses, similar to all the docked synaptic vesicles previously examined at resting active zones of the junctions [45]. Furthermore, the average contact area of the hemifused synaptic vesicles was found to be more than two-fold greater than that of the docked synaptic vesicles without hemifusion. The results demonstrate that synaptic vesicles at the active zone, if not all, undergo hemifusion preceding fusion with the presynaptic membrane during synaptic activity, which is consistent with other studies [34,35]. However, the rarity of hemifused synaptic vesicles at active zones indicates that hemifusion vesicles are at unstable states between docking and fusion. The nanometer-scale quantification of the spatial relationship of synaptic vesicles with the presynaptic membrane by electron tomography may provide further understanding about the synaptic vesicle fusion pathway.

## 2. Results

Electron tomography on a tissue section relies on multiple 2-dimensional (2D) transmission electron microscope images of the section, collected at different tilt angles to generate a 3-dimensional (3D) reconstruction of the section. The relationships of structures within the reconstructed volume can be studied at 2–3 nanometer (nm) spatial resolution by examining the structures of interest in serial virtual slices made through the volume, which are much thinner than the tissue section, and their 3D surface models can be generated by rendering segmented volumes of interest that enclose the structures of interest [48]. Using electron tomography, 34 reconstructed volumes of randomly selected axon terminals of frog NMJs fixed during evoked synaptic activity were generated, and more than 80 synaptic vesicles at active zones of the NMJs were examined, using serial virtual slices (<1 nm thick) through the volume reconstructions.

**Figure 1:**
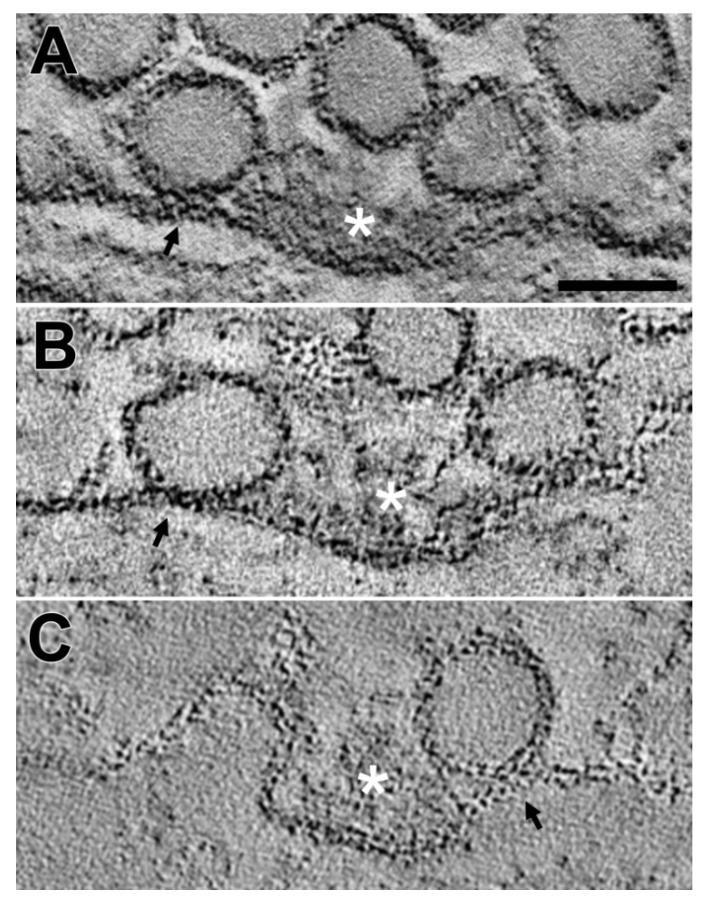
Three docked synaptic vesicles at three different active zones of axon terminals in frog neuromuscular junctions (NMJs), fixed during repetitive electrical nerve stimulation at 10 Hz, evoking synaptic activity. Virtual slices (3.5 nm thick) from three different reconstruction volumes show activated active zones, sectioned nearly in the transverse plane of the active zones. (**A**) The membrane of a synaptic vesicle at an active zone is apposed onto the presynaptic membrane, without any significant merging or gap between the membranes (a black arrow). Opposite to the docked synaptic vesicle, the active zone also has a synaptic vesicle close to the presynaptic membrane. At the active zone, marked by a white asterisk, the densely stained AZM macromolecules are connected to both of the synaptic vesicles. Scale bar = 50 nm. (**B**) A synaptic vesicle at an active zone, which was previously analyzed (See Figure 2E in [45]) is docked with the presynaptic membrane and shows a significant merging with the presynaptic membrane (a black arrow), indicating that the vesicle is hemifused with the presynaptic membrane. Note that the hemifused synaptic vesicle is also connected to the AZM macromolecules. (**C**) A synaptic vesicle at an active zone is docked with the presynaptic membrane, without any significant merging or gap between the membranes (a black arrow). Opposite to the docked synaptic vesicle, the active zone also has a synaptic vesicle completely fusing with the presynaptic membrane. Similar to Figure 2A,B, the AZM macromolecules at the active zone (a white asterisk) are connected to the docked synaptic vesicle and the fusing synaptic vesicle.

Serial virtual slices through the reconstructions helped identify that the active zones of axon terminals contained synaptic vesicles, docked or fused (Figure 1). The synaptic vesicles in contact with the presynaptic membrane at active zones are commonly observed to be docked with the presynaptic membrane without notable merging between the vesicle membrane and the presynaptic membrane (Figure 1A,B). Synaptic vesicles fusing with the presynaptic membrane are also observed at active zones (Figure 1C), consistent with other studies using conventional 2D electron microscopy and 3D electron tomography [3,47]. The docked synaptic vesicles (Figure 1A,B) show a direct connection with the densely stained AZM macromolecules [43,44,47,49,50,51], and the fused synaptic vesicle in Figure 1B is also connected to AZM macromolecules, indicating that fused synaptic vesicles result from docked synaptic vesicles [47]. Furthermore, a docked synaptic vesicle in Figure 1B shows its merging with the presynaptic membrane such that a portion of their contact site looks similar to a single membrane, indicating that the vesicle is hemifused with the presynaptic membrane. The same vesicle also shows its direct connection with the AZM macromolecules, consistent with previous findings that all docked synaptic vesicles are connected to the AZM macromolecules [49,50], and other studies [34,35] suggesting that any docked synaptic vesicle can be hemifused with the presynaptic membrane at the active zone before fully fusing with the presynaptic membrane.

**Figure 2:**
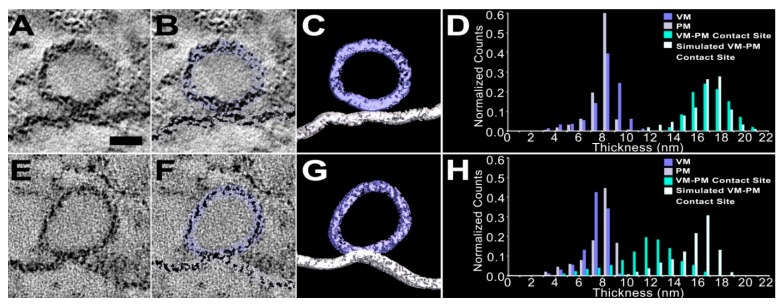
Vesicle membrane–presynaptic membrane (VM–PM) contact sites of two docked synaptic vesicles on the presynaptic membrane, at two different active zones and measured membrane thicknesses. (**A**,**E**) Virtual slices (0.5 nm thick) of the two docked synaptic vesicles on their presynaptic membranes at active zones of the frog NMJs. Scale bar = 25 nm. (**B**,**F**) Surface models of the two docked synaptic vesicles (light blue) and the stretch of presynaptic membranes (light gray) are superimposed on the two virtual slices. (**C**,**G**) Portions of the models (10 nm thick) of the vesicles and presynaptic membranes are visualized. The vertices used to generate the models provide continuous surfaces for the vesicle membrane and presynaptic membrane, while accounting for irregularities in the membrane staining. (**D**,**H**) Frequency distributions of thousands of vertex-to-vertex thickness measurements made across the entire membranes of the two docked synaptic vesicles and the presynaptic membranes, from their 3D surface models. For the docked synaptic vesicle in **A**–**C**, the membrane thicknesses of the individual membranes away from their contact sites are 8.3 nm ± 1.5 nm (mean ± SD) for the VM and 8.1 nm ± 1.1 nm (mean ± SD) for the PM, and the combined thickness of the vesicle membrane and presynaptic membrane at the contact site (16.4 nm ± 1.5 nm; mean ± SD) is not different from the expected combined thickness (16.5 nm ± 1.9 nm; mean ± SD) obtained by summing randomly the vesicle membrane and presynaptic membrane thicknesses away from the contact site (Bootstrap test, *p* = 0.97; See Materials and Methods). For the docked synaptic vesicle in **E**–**G**, the VM and the PM are 7.5 nm ± 1.0 nm and 7.8 nm ± 1.5 nm, respectively. The combined thickness of the vesicle membrane and presynaptic membrane at the contact site (11.6 nm ± 2.4 nm; mean ± SD) is less than its expected combined thickness (Bootstrap test, *p* < 0.05), showing that the docked vesicle is merged with the presynaptic membrane. Note that each membrane varies in thickness from one vertex to another, mainly due to irregular staining of the membrane.

To quantitatively characterize the spatial relationship of the vesicle membrane to the presynaptic membrane at the contact sites, their 3D surface models were used (Figure 2C,G). The surface models were generated using previously published methods to reliably delineate the contrast boundaries of the membranes and to provide nearly accurate 3D representation of the vesicles and the presynaptic membranes [48,52]. Here, surface models of 81 docked synaptic vesicles and 34 presynaptic membranes were used to obtain the quantitative measurement of their membrane thicknesses, because it is difficult to accurately measure each membrane thickness by manual measurement when relying on individual virtual slices, mainly due to generally varying membrane curvature and irregular membrane staining. The membrane thicknesses of the synaptic vesicles and the presynaptic membranes away from their contact sites were measured using an algorithm (see Materials and Methods and also [45]), by calculating the shortest distances across the membrane between vertices that established the membrane surfaces (Figure 2B,F). The combined membrane thickness at each of the contact sites was measured similarly (Figure 2B,F) instead of individual membrane thicknesses, because the relationship of the two membranes at the interface could not be reliably determined due to the proximity of the membranes compared to the 2–3 nm spatial resolution and the variation in the staining pattern of the membranes at the interface. The combined thickness at the vesicle membrane–presynaptic membrane contact site was compared to the thickness of each membrane beyond the contact site, using the expected combined membrane thickness at the contact site (Figure 2; See Materials and Methods and also [45]). For 74 vesicles out of 81 docked vesicles, the average thickness for the vesicle membrane away from the contact site was 7.9 nm ± 0.61 nm (mean ± SD), while for 34 presynaptic membranes beyond the contact site, the average thickness was 7.8 nm ± 0.71 nm (mean ± SD)—consistent with membrane thickness measurements made by others on 2D electron microscope images of cellular membranes in tissue sections [53,54]. The average thickness of the combined vesicle membrane and presynaptic membrane at the site of contact for the same docked vesicles, i.e., from the luminal surface of the vesicle membrane to the extracellular surface of the presynaptic membrane, was 16.1 nm ± 1.3 nm (mean ± SD). Each of the combined membrane thicknesses was not different from the sum of the average inter-surface distance of the two membranes beyond their contact site (Bootstrap test, *p* > 0.1). Thus, at vesicle membrane–presynaptic membrane contact sites there is no detectable gap between or merging of the two membranes at the spatial resolution of 2–3 nm [48], which agrees well with a recent study showing that all docked synaptic vesicles at active zones of resting axon terminals in frog NMJs are in contact with the presynaptic membrane without any notable merging or gap [45].

Most of the docked synaptic vesicles with the presynaptic membrane at active zones displayed no notable merging or gap with it, but a small portion of the docked vesicles (*n* = 7) were discovered to be hemifused with the presynaptic membrane (Figure 2B,E). Using their surface models (Figure 2C,G), the combined thickness of the two membranes at their contact site can be compared to the sum of their membrane thicknesses beyond the contact site. For the hemifused vesicles, the average thickness of the vesicle membrane away from the contact site was 7.8 nm ± 0.26 nm (mean ± SD) and the membrane thickness of the presynaptic membrane away from their contact site was 7.7 nm ± 0.30 nm (mean ± SD)—consistent with the measurements of the docked synaptic vesicles without hemifusion. However, in contrast to the docked vesicles without hemifusion, the average thickness of the combined vesicle membrane and presynaptic membrane at the contact site for each of the hemifused vesicles was 11.6 nm ± 0.70 nm (mean ± SD), which was significantly less than the sum of the thicknesses of the two membranes beyond the contact site (15.8 nm ± 0.24 nm (mean ± SD); Mann–Whitney U test, *p* < 0.05; see also [45]). The results demonstrate that at the active zone of axon terminals in frog NMJs, fixed during evoked synaptic activity, a small portion of the docked synaptic vesicles show detectable merging of the two membranes at their vesicle membrane–presynaptic membrane contact sites, providing evidence that docked synaptic vesicles undergo hemifusion at the active zone.

**Figure 3:**
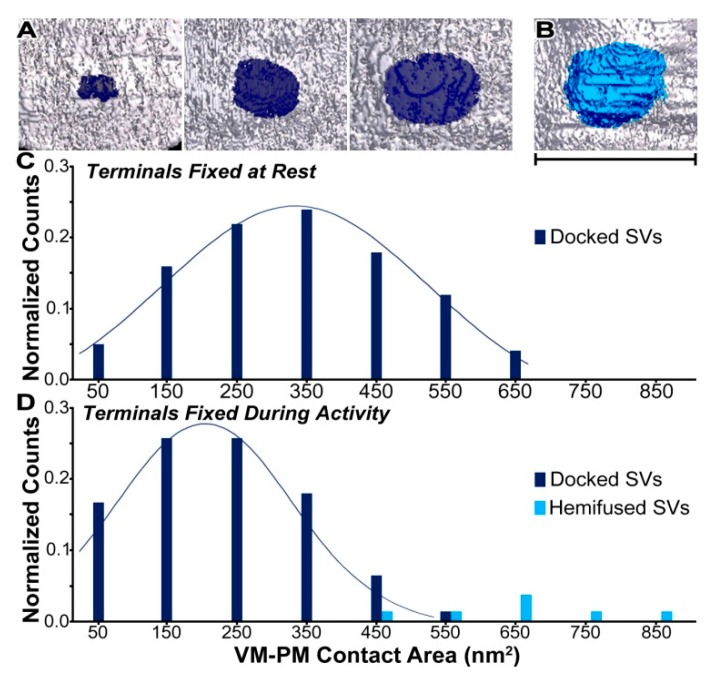
Vesicle membrane–presynaptic membrane (VM–PM) contact areas of docked synaptic vesicles (SVs) at the active zones, fixed at rest and during the repetitive electrical nerve stimulation at 10 Hz. (**A**) The VM–PM contact areas for three docked SVs (dark blue) in terminals fixed at rest were mapped on surface models of the PM, from the cytoplasmic side of each of the active zone. The extents of the marked contact sites of the vesicles show that their contact areas are different in size. Based on the number of pixels within the perimeter of the contact sites projected onto best-fit planes, the VM–PM contact areas (left, middle, and right) were 60 nm^2^, 300 nm^2^, and 600 nm^2^, respectively. (**B**) The contact site of a docked synaptic vesicle hemifused with the presynaptic membrane was mapped on the surface model of the presynaptic membrane (teal-blue). The extent of the contact area is 630 nm^2^. Scale bar is 50 nm for **A** and **B**. (**C**) The histogram of the VM–PM contact areas of the 101 docked SVs, measured as in **A**, in terminals fixed at rest. The area varies more than 13-fold, with a normal distribution across the population (330 ± 150 nm^2^; mean ± SD). The bin size is 100 nm^2^. (**D**) The histograms of the contact areas of the 74 docked synaptic vesicles without hemifusion (dark blue) and seven hemifused synaptic vesicles (cyan). The average contact area and standard deviation of the docked vesicles without hemifusion is 220 nm^2^ ± 120 nm^2^ (mean ± SD). The average contact area of the hemifused synaptic vesicles (660 nm^2^ ± 150 nm^2^; mean ± SD) is greater than the average area of the docked synaptic vesicles without hemifusion (Mann–Whitney U test, *p* < 0.01). Adapted from Jung et al. 2016 [45].

When the contact sites were mapped onto surface models of the vesicle membranes or presynaptic membranes, the areas were generally oval, although there were occasionally small irregularities in their perimeter due to the irregular staining (Figure 3A,B). The contact area, i.e., the lateral extent of a contact site, was measured by projecting the contact site onto a best-fit plane, which compensated for small irregularities in the surface models of the membranes (Figure 3A,B; see Materials and Methods and also [45,51]). From vesicle to vesicle, the contact area varied more than 15-fold, from ~50 nm^2^ to ~850 nm^2^ (270 nm^2^ ± 180 nm^2^, mean ± SD) (Figure 3). However, the distribution of the contact area was different from that of the active zones at the frog NMJs fixed at rest (Figure 3; See also [45]). Furthermore, when the docked synaptic vesicles were separated into two populations—docked vesicles without hemifusion and hemifused vesicles—the contact area of the docked vesicles without hemifusion varied more than 10-fold, from ~50 nm^2^ to ~550 nm^2^, with a normal unimodal frequency distribution (normality test, *p* = 0.9), and the contact area of the hemifused vesicles (*n* = 7) varied from ~450 nm^2^ to ~850 nm^2^ (Figure 3D). Even though this should be interpreted cautiously, as the number of the hemifused synaptic vesicles is small, the statistical comparison indicates that the average contact area of the seven hemifused synaptic vesicles (660 nm^2^ ± 150 nm^2^, mean ± SD) is different from that of the 74 docked synaptic vesicles (230 nm^2^ ± 120 nm^2^, mean ± SD; Mann–Whitney U test, *p* < 0.01), demonstrating that the contact area of docked vesicles increases as they become hemifused with the presynaptic membrane.

Altogether, the results show that at the spatial resolution of electron tomography (2–3 nm), a small portion of docked synaptic vesicles at active zones of frog NMJs, fixed during evoked synaptic activity, are hemifused with the presynaptic membrane, while the rest of the docked synaptic vesicles are in contact with the presynaptic membrane without such hemifusion (Figure 1 and Figure 2), and the hemifused vesicles tend to have larger contact areas than the docked vesicles without hemifusion.

## 3. Discussion

Electron tomography on axon terminals of frog NMJs chemically fixed during synaptic activity demonstrated that a small portion of synaptic vesicles are hemifused with the presynaptic membrane at the active zones; seven hemifused synaptic vesicles out of 81 synaptic vesicles in contact with the presynaptic membrane were found at active zones from 34 reconstructions. The measured combined membrane thickness of each of the hemifused synaptic vesicles was less than the sum of the individual synaptic vesicle and presynaptic membrane thicknesses, in contrast to the 74 docked synaptic vesicles without such shift in the combined membrane thickness toward a single membrane thickness. The contact area of the hemifused vesicles is more than two-fold greater on average than that of the docked vesicles, suggesting that the contact area increases as a docked vesicle becomes hemifused with the presynaptic membrane.

**Figure 4:**
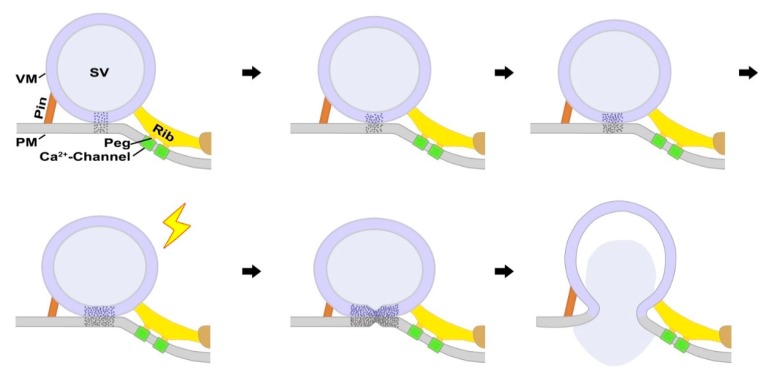
Sequential stages of a synaptic vesicle leading to the formation of the fusion pore through hemifusion for the neurotransmitter release at the active zone of axon terminals of frog NMJs. The vesicle membrane (VM) of a synaptic vesicle (SV) is in contact with the presynaptic membrane (PM) at the active zone of the frog NMJ (indicated by stipples). The docked vesicle is connected to several AZM components, such as ribs and pins. Ribs link the vesicle membrane to the presynaptic membrane via pegs, which are connected to PM macromolecules thought to include Ca^2+^ channels (See Jung et al. [45]). The vesicle membrane of the docked vesicle increases its contact area with the presynaptic membrane, by the shortening of its AZM components directly linking the vesicle membrane to the presynaptic membrane, without detectable merging between the membranes. The further shortening of the AZM components increases the contact area more. When an action potential arrives at the active zone, the vesicle membrane and the presynaptic membrane are hemifused, enhancing the contact area further by the attractive force generated by the greater shortening of the AZM components, leading to the formation of an irreversible fusion pore. As the pore expands, releasing the neurotransmitter contained in the vesicle for synaptic transmission, the vesicle membrane flattens into the presynaptic membrane.

Studies using protein-free lipid membranes or liposomes reconstituted with SNARE proteins provided reliable evidence that the hemifusion state is present in the pathway of membrane fusion and it can be stable [26,55,56,57,58,59]. Consistently, electron tomography studies on brain slices of rats chemically fixed by perfusion reported that ~74% of docked synaptic vesicles are hemifused, suggesting that they may represent the immediately releasable pool of synaptic vesicles upon stimulation [35,60]. Here, hemifused synaptic vesicles were observed among docked vesicles at active zones of axon terminals in frog NMJs, fixed during synaptic activity, however, it was found that more than 90% of the docked vesicles have no indication of hemifusion. Consistently, several other recent electron tomography studies on hippocampal slices of rats and mice, in central nervous systems fixed by high-pressure freezing, have not reported any observation of hemifused synaptic vesicles [43,44]. There were also no hemifused synaptic vesicles found at active zones of resting axon terminals of frog NMJs prepared by chemical fixation [45], which does not agree with the expected numerous hemifused vesicles at active zones that are thought to be induced by chemical fixation [44]. Thus, although hemifusion of synaptic vesicles is likely to occur at active zones prior to fusion, as a part of productive fusion pathways, the results shown here and from other studies using chemical fixation or high-pressure freezing [43,44,45,61] indicate that hemifused vesicles are not commonly observable, probably due to their instability, which facilitates full fusion with the presynaptic membrane. The occurrence and stability of hemifused synaptic vesicles may depend on synapse specific factors regulating the spatial relationship of synaptic vesicles with the presynaptic membrane at the active zone, such as multiple AZM macromolecules connected to docked synaptic vesicles, which may contain key proteins for fusion of the vesicles with the presynaptic membrane [34,35,45,47,49,50,62,63,64]. Accordingly, docked synaptic vesicles are likely to constitute immediately release-ready vesicles in general [43,44]. However, only 1–3% of the docked synaptic vesicles at the resting frog NMJs are known to fuse with the presynaptic membrane when a nerve impulse arrives [7,65], and the common presence of docked synaptic vesicles at active zones of axon terminals in frog NMJs fixed during synaptic activity supports that only a portion of the docked vesicles at the active zone immediately fuse with the presynaptic membrane. Accordingly, if the vesicles undergo hemifusion prior to fusion, the portion of the hemifused synaptic vesicles is expected to be small at frog NMJs. Consistently, the portion of the hemifused vesicles from the docked synaptic vesicles at active zones of the frog NMJs fixed during synaptic activity is small (<9%). According to fusion-through-hemifusion models, the hemifused region at the contact site of a docked vesicle may expand to promote the formation of a fusion pore within it, or may be able to form a fusion pore without such expansion [33]. The measurement of the contact areas of docked synaptic vesicles at active zones revealed that the average contact area of the hemifused synaptic vesicles is greater than that of the docked synaptic vesicles by more than two-fold, suggesting that the hemifused synaptic vesicles expand their contact area with the presynaptic membrane, promoting the formation of a fusion pore. This is probably by the force generated from the formation of SNARE complexes and other auxiliary proteins that are likely to be contained in multiple AZM macromolecules connected to the vesicles near their contact site with the presynaptic membrane [41,45,49,51,63,64]. Altogether, the results shown here demonstrate that the hemifusion of synaptic vesicles occurs after a nerve impulse arrives at the active zones of axon terminals of the frog NMJ, providing evidence that the hemifusion is an intermediate state for fusion of synaptic vesicles at the active zone and the synaptic vesicles proceed to hemifusion for full fusion with the presynaptic membrane (Figure 4). The findings also suggest that a high-resolution quantitative approach combined with electron tomography may help characterize the fusion pathways that synaptic vesicles undergo at the active zone and provide further understanding of synaptic vesicle fusion with the presynaptic membrane critical for synaptic transmission.

## 4. Materials and Methods

### 4.1. Tissue Preparation

Details of the tissue preparation of synapses on skeletal muscles of frogs can be found elsewhere [45,47]. Briefly, paired cutaneous pectoris muscles of two adult Rana pipiens (5 cm nose rump length, male), obtained in summer (Hazen JM Frog Co., Alburg, VT, USA) and sacrificed by double-pithing, were pinned out in a Petri dish containing Ringer’s solution (115 mM NaCl, 2 mM KCl, 2.2 mM CaCl_2_, 1 mM NaHPO_4_⋅H_2_O; 220–230 milliosmoles (mOsM), pH 7.2). The cut end of the nerve was drawn into a suction electrode. Stimulation parameters were established by passing single current pulses through the suction electrode, while using a dissecting microscope to monitor muscle contractions induced by synaptic transmission. The threshold for contraction of the entire muscle occurred at a current amplitude of ~1 μA for 1 msec. The Ringer’s solution was then replaced for more than 5 min by Ringer’s solution containing 10^−5^ g/mL (+)-Tubocurarine chloride hydrate (Sigma-Aldrich, Inc., St. Louis, MO, USA) to block muscle contractions. The (+)-Tubocurarine-containing Ringer’s solution was replaced by Ringer’s solution containing 0.8–1% glutaraldehyde (220–230 mOsM total; pH 7.2), as the resting terminals that were not electrically stimulated were fixed in previous studies, and nerve stimulation simultaneously began at 10 Hz with a current amplitude 10 times greater than threshold. Stimulation continued for 2 min; it was previously observed under a dissecting microscope that in stimulated nerve–muscle preparations not exposed to (+)-Tubocurarine, contractions of all muscle fibers ceased after 2 min in the fixative, indicating all of the NMJ’s were fixed at that time. After stimulation, the muscles remained in fixative for 40 min. They were further processed at room temperature for electron tomography, according to the method used for preparing resting terminals. They were further fixed and stained for 1 h in 1% OsO_4_ in phosphate buffer (220–230 mOsM total, pH 7.2), washed for one hour in H_2_O, stained one hour in saturated aqueous uranyl acetate, dehydrated in increasing concentrations of ethanol, and embedded flat in a 1-mm-thick wafer of Eponate 12 (Ted Pella). The animal experimentation described here was approved by Stanford University’s (Protocol Number 10505, 24 January 2008) and Texas A&M University (AUP Number 2011-8, 23 May 2011) administrative panels on laboratory animal care (IACUC), which oversee the use of animals according to U.S. federal regulations.

### 4.2. Sections

The thickness of the tissue sections varied from 50 nm to 150 nm, based on measurements from the reconstructed volumes. They were stained for 10 min with saturated uranyl acetate in methanol, rinsed with water, stained again with Reynolds lead citrate for 10 min, and again rinsed with water.

### 4.3. Data Collection

34 datasets from terminals fixed during evoked impulse activity with gluteraldehyde were collected at a magnification ranging from 59,000× to 125,000×, using one of two electron microscopes designed for automatic data acquisition: 1) FEI TF30 Polara electron microscope (FEI Company Hillsboro, OR, USA) equipped with a 2048 × 2048 Tietz TemCam-F224HD CCD (Tietz Video and Imaging Processing Systems GmbH, Gauting, Germany); and 2) FEI Tecnai G2 F20 electron microscope (FEI Company Hillsboro, OR, USA) equipped with a 2048 × 2048 Gatan CCD (Gatan, Inc., Pleasanton, CA, USA). The stage on each microscope was cooled to liquid nitrogen temperature to reduce beam damage to the specimen. Datasets consisted of images taken at 1° tilt intervals to ± 60° along a single tilt axis or ± 60° along two orthogonal tilt axes.

### 4.4. Reconstruction

The tilt-images were aligned automatically using 5 or 10 nm gold colloid (British Biocell International, Cardiff, UK) deposited on one or both sides of the sections as fiducial markers before data collection. For the datasets used in the current study, the scheme provided an average accuracy less than 1.5 pixels (root mean square) or 0.60 nm (root mean square). The reconstructions were made by a weighted back-projection method. Both the alignment and reconstruction algorithms were in the unified software package for electron tomography, EM3D (www.em3d.org) [48].

### 4.5. Virtual Slices, Segmentations, and Surface Models

Virtual slices through the reconstructed tissue sections were one voxel thick. Depending on the magnification of the dataset, the virtual slice thickness represented 0.43 nm to 0.58 nm of a tissue section’s thickness. When necessary, the angular orientation of the slice plane was adjusted to maximize contrast boundary discrimination of the structures under study.

Structures were segmented from the reconstructions using a combination of manual and semi-automatic methods in EM3D to define individual volumes of interest (VOIs). For the presynaptic membranes and vesicle membranes, which were heavily stained and had a simple geometry, a semi-automatic scheme was used and manually adjusted as necessary. The VOIs were slightly larger than the structures that they enclosed, to allow accurate and complete isodensity-surface calculations for the surface models. EM3D was used to render a surface model from each VOI. The rendering was done based on gray scale values of each VOI. The surface model of each VOI, such as a vesicle membrane and presynaptic membrane, was generated and its isodensity value was adjusted to provide nearly the best fit to the contrast boundary of the VOI [48].

### 4.6. Measurements

#### 4.6.1. Membrane Thickness

For docked synaptic vesicles, thickness measurements were made on separate surface models of the vesicle membrane and presynaptic membrane, away from the vesicle membrane–presynaptic membrane contact site and the combined vesicle membrane–presynaptic membrane at the contact site, using an algorithm designed to calculate the shortest distances across the membrane between vertices that established the membrane surfaces, as described in a previous study [45]. Specifically, the membrane thickness measurements were made for all vertices of each surface model. For each vertex, straight lines were drawn that connected it to each of its neighboring vertices and extended beyond the surface model. Along the direction of each line, and within a cylinder with a radius of 1.5 nm or less, the distance between the two farthest vertices was measured. The minimum value of the measured distances along the cylinders from a vertex to all of its neighboring vertices was determined as the membrane thickness at the position of that vertex.

#### 4.6.2. Extent of the Vesicle Membrane–Presynaptic Membrane Contact Area.

The vertices at the interface of the vesicle membrane (VM) and presynaptic membrane (PM) at their contact site were projected onto the best-fit plane, which compensated for small irregularities in the surface models of the membranes, along an eigenvector containing the least eigenvalue, which was calculated using the covariance matrix of the vertices’ coordinates, as described in previous studies [45,51]. The best-fit plane was pixilated, and each pixel was standardized to have the area of one face of a voxel from the reconstruction, to maintain scale. The contact area was calculated by counting the number of pixels, typically less than 1 nm^2^ in size, that contained the projected vertices, and converting it to area according to scale.

### 4.7. Statistical Analyses

Kolmogorov–Smirnov tests for normality and Mann–Whitney U tests for comparing two population means were performed with the OriginPro software package (version 8, OriginLab, Northampton, MA, USA).

The bootstrap method [66], which was performed with the IDL software package (version 7 Exelis, Boulder, CO, USA), was applied to test whether the combined thickness at the vesicle membrane–presynaptic membrane contact site was different from the sum of the vesicle membrane and presynaptic membrane thicknesses away from the contact site for each synaptic vesicle, as described in a previous study [45]. Accordingly, the distribution of the actual thickness measurements across the vesicle membrane–presynaptic membrane contact site (*T_m_*) was compared to simulated distributions of the sum of vesicle membrane and presynaptic membrane thicknesses (*T_i_*). For simulated distributions, randomly selected vertex–vertex thickness measurements of the SV membrane were added to randomly selected vertex–vertex thickness measurements of the PM, using 2000 iterations of this calculation. *p*-values were calculated and averaged from 10,000 iterations of simulated distributions, according to the following equation (Equation (1)):

(1) $$p=\frac{\sum_{i}probability\;that\left|{\bar{T}}_{i}-T_{i}\right|\geq \left|{\bar{T}}_{i}-{\bar{T}}_{m}\right|}{10000}$$

where i∈[1,10,000], $\bar{T_{m}}$ is the mean of *T_m_*, and $\bar{T_{i}}$ is the mean of *T_i_*.

Figure layouts: Figure layouts were prepared using Adobe Photoshop CS3 (Adobe Systems, San Jose, CA, USA).

Computer hardware and software: For the analyses, PC computers were used loaded with Windows Vista or Windows 7, IDL (Interactive Data Language) version 7, Java 6, 7, or 8, EM3D (version 1.3) coded in IDL, and EM3D 2.0 coded in C^++^ and Java.
